# Personal and Environmental Contributors to Sedentary Behavior of Older Adults in Independent and Assisted Living Facilities

**DOI:** 10.3390/ijerph17176415

**Published:** 2020-09-03

**Authors:** Mary P. Kotlarczyk, Andrea L. Hergenroeder, Bethany Barone Gibbs, Flor de Abril Cameron, Megan E. Hamm, Jennifer S. Brach

**Affiliations:** 1 Division of Geriatric Medicine, Department of Medicine, University of Pittsburgh, Pittsburgh, PA 15260, USA; 2 Department of Physical Therapy, University of Pittsburgh, Pittsburgh, PA 15260, USA; alocke@pitt.edu (A.L.H.); jbrach@pitt.edu (J.S.B.); 3 Department of Health and Human Development, University of Pittsburgh, Pittsburgh, PA 15260, USA; bbarone@pitt.edu; 4 Division of General Internal Medicine, Department of Medicine, University of Pittsburgh, Pittsburgh, PA 15260, USA; flg9@pitt.edu (F.d.A.C.); mehst52@pitt.edu (M.E.H.)

**Keywords:** sedentary behavior, older adults, residential care, focus groups, thematic analysis, qualitative research

## Abstract

Sedentary behavior is associated with negative health outcomes and unhealthy aging. Older adults are the most sedentary age group, and decreasing sitting time represents an intervention target for improving health. Determinants of sedentary behavior have been examined in older adults living in their own homes, yet less is known about sedentary behavior of older adults in residential care facilities. The purpose of this study was to explore factors contributing to sedentary behavior among residents of independent and assisted living facilities. We conducted eight focus groups with residents (*n* = 44) and semi-structured interviews with staff (*n* = 6) across four living facilities. Audio recordings were transcribed and analyzed using an iterative, inductive approach. Three salient themes were identified. Residents and staff both viewed sedentary behavior negatively unless it was in the context of social engagement. Additionally, fear of falling was discussed as a significant contributor to sedentary behavior. Finally, residents felt the community living environment contributed to their sedentary behavior while staff did not. Our findings provide valuable insight for designing targeted interventions for older adults in residential facilities and suggest thinking beyond the individual and considering environmental influences on sedentary behavior in the residential care setting.

## 1. Introduction

Older adults are the most sedentary age group, spending an estimated 9.4 h per day engaged in low energy seated activities [1]. Sedentary behavior is defined as any waking activity using less than 1.5 metabolic equivalents (METs) while in a seated, reclining, or lying position [2]. Engagement in high levels of sedentary behavior is associated with poor health outcomes including cardiometabolic disease [3], sarcopenia [4], and increased risk of mortality [5,6,7,8]. Several studies have found these health risks to be independent of time spent engaged in physical activity, distinguishing sedentary behavior as an important determinant of health outcomes [3,6,9].

Given the detrimental effects of sedentary behavior and the high levels of sedentary time in older adults, there is a need to improve our understanding of factors influencing sedentary behavior in the aging population. Gaining knowledge of these behavioral determinants is necessary to develop targeted interventions to reduce sedentary behavior. Cross-sectional and longitudinal studies have found factors correlated with sedentary behavior such as age, gender, income, and health status [10]. There is a recognized need for more qualitative research studies to better understand older adults’ perceptions and beliefs about sedentary behavior and the contexts around where, when, and why sedentary behavior most occurs in order to design effective interventions [10,11]. A recent systematic review and thematic analysis of 15 qualitative studies examining sedentary behavior in older adults found several key areas of focus pertinent to intervention development, namely, the need to address knowledge about sedentary behavior and its relationship to health, the integrated nature of sedentary activities into daily life, enjoyment of sedentary activities, and perceptions about sedentary behavior with age [12]. While this information is essential for designing interventions for older adult populations, all studies reviewed focused on community-dwelling older adults or specific patient populations (e.g., cardiac rehabilitation). None considered sedentary behavior in the context of residential communities.

Determinants of sedentary behavior are likely to exist on multiple levels of the Social Ecological Model and to differ across population groups and environments [11,13,14]. The ecological perspective is grounded in the notion that health behaviors are not solely the result of an individual’s beliefs, attitudes, and abilities. Rather, people are part of a larger environment of policies, space, and social norms, and health behaviors are shaped by this surrounding landscape [11,15]. Understanding determinants of health behavior across these multiple levels of influence is important for designing effective interventions to change behavior patterns. Older adults in residential communities such as independent and assisted living facilities (ILFs and ALFs) have a different living environment and may be subject to unique social and environmental influences on sedentary behavior; therefore, this has been identified by expert consensus as a priority area of research [13,16,17]. Our previous work has shown that older adults in residential facilities are more sedentary than those who live in their own homes [18]. The purpose of this study was to explore factors contributing to sedentary behavior among older adults residing in ILF and ALF communities. The Social Ecological Model emphasizes the influence of interpersonal relationships and the environment on individual health behaviors, and previous work has indicated a role for family and caregivers in the sedentary behavior of older adults [19,20]. Therefore, we also sought to understand caregiver perspectives on the sedentary behavior of residents.

## 2. Materials and Methods

### 2.1. Study Design and Setting

We conducted focus groups with residents at two ILFs and two ALFs as well as semi-structured interviews with facility staff. We purposely selected residential communities that provided varying levels of care for older adults with a range of physical abilities. Living facilities were part of a larger system of senior communities in the greater area surrounding Pittsburgh, Pennsylvania, United States. All facilities were multistory buildings with common social areas, communal dining rooms, and onsite staff such as a manager, front desk attendant/concierge, activities director, food servers, and housekeepers. Residents had private rooms with a small kitchen, bathroom, living area, and optional additional bedrooms. ILFs and ALFs differed in the extent of support offered to residents. Independent facilities provided no routine health care services or support with daily activities, although residents could receive additional care and assistance from outside service agencies if desired. Assisted living communities provided onsite medical staff and assistance with activities of daily living including medication administration, bathing, dressing, and ambulation.

### 2.2. Ethical Considerations

The University of Pittsburgh Institutional Review Board reviewed the study protocol (PRO17020536) and determined it met the exemption criteria for minimal risk research. Prior to commencement of all focus groups and interviews, participants were made aware of their right to withdraw from the study at any time, potential risks to privacy, and steps to safeguard information including the removal of identifiable information from transcripts. All participants were notified that audio recordings were being made of focus group conversations and interviews.

### 2.3. Participants and Recruitment

Residents age 65 years and older were recruited to participate in focus group discussions. Recruitment strategies included announcements at facility mealtimes, informational sessions at tables in community areas, and advertisements posted within the facility. We conducted eight focus groups to be reasonably assured of capturing sufficient data to reach thematic saturation, when no new themes emerge from the data [21].

We conducted individual interviews with staff members at the same facilities where resident focus groups were held. Members of the research team met with facility administrators and talked with staff about the study. Advertisements were also placed in staff areas. We intended to conduct 20 interviews with facility staff members in order to be reasonably assured of reaching thematic saturation [22]. However, recruitment efforts had a low response, and we were unable to recruit more than six interview participants.

### 2.4. Data Collection

Two focus groups were held on site at each of the four living facilities. Focus groups were conducted during the summer and fall of 2017. The qualitative methodologist or a trained research assistant moderated each focus group, with the principal investigator or a research assistant serving as a notetaker. The qualitative methodologist had extensive experience conducting focus groups; two research assistants served as additional moderators, both of whom had been trained by the qualitative methodologist and had prior experience moderating focus groups. Moderators followed a focus group guide developed by the research team to explore multiple levels of the social ecological model (Supplemental File S1). Topics for discussion included residents’ behaviors and activities in the facility; knowledge, attitudes, and beliefs about sedentary behavior; if and how their living space influenced sitting; and barriers to reducing sitting time. Moderators attempted to facilitate discussion in such a way that all participants provided input. Focus groups consisted of 4–7 participants per group and lasted 47–60 min (mean duration 54 min). Facility staff were interviewed individually via telephone by an experienced research assistant. Interviews lasted an average of 22 min (range 14–31). Interview topics were similar to the topics addressed in the focus groups, although questions were about residents rather than the interviewed individuals (Supplemental File S2). Focus group and interview participants also provided basic demographic information.

### 2.5. Data Analysis

Analysis was conducted following Braun and Clarke’s description of Thematic Analysis [23], in which researchers familiarize themselves with the transcribed data, organize it by assigning relevant tags to the segments of the transcripts through a process known as “coding,” and then combine the information from those codes into “themes” that summarize the data as a whole. Focus group discussions and interviews were audio recorded and transcribed. Anonymized transcripts were imported into Atlas.ti 8 software (Atlas.ti GmbH, Berlin, Germany) for coding and data management. Separate codebooks for the interviews and focus groups were generated by the research assistants who had conducted the interviews and focus groups, using an iterative, inductive approach known as “editing” to determine codes representing appropriate topics and emerging themes arising from the transcripts [24]. In this approach, codes are not developed according to what might be expected from the literature, but instead generated from the content of the transcripts. The qualitative methodologist and principal investigator reviewed preliminary codebooks to check for clarity of code definition and representation of topics discussed. After finalized codebooks were agreed upon, all transcripts were independently coded by the principal investigator and a research assistant. The two coders then met to compare coding and resolve any discrepancies through discussion, thus ensuring a consistent application of codes across the transcripts. Following the iterative coding process and adjudication of coding discrepancies, the principal investigator produced themes describing patterns or commonalities in the data related to influences on sedentary behavior. For something to be considered a theme, it had to represent the majority viewpoint across the focus groups or interviews. Resulting themes were discussed with the research assistant who completed coding, the qualitative methodologist, and all other co-investigators as a form of investigator triangulation [25].

## 3. Results

### 3.1. Participant Characterisitcs

We recruited 44 older adults for focus group participation (Table 1). Participants across the facility types were of similar age with a mean age of 86 years (range 65–97). The majority of participants were women (70.5%), white (95.4%), and widowed (65.9%)—reflective of the facility populations. The majority of older adults at both types of facilities used an assistive device with mobility aid use being more prevalent among those in ALFs. Staff participants were women ranging in age from 18–63 years (mean age 41 years). Staff experience in the residential care setting ranged from 1–6 years. Participating staff included housekeepers, food service attendants, front desk concierge, and activities directors.

### 3.2. Thematic Analysis

We stratified focus groups by living facility type to allow for varying perspectives on sedentary behavior among residents from the two types of living environments. However, during analysis, we found similar themes emerge from focus groups discussions regardless of facility type. We uncovered three common themes pertaining to sedentary behavior of older adults in ILFs and ALFs. The first theme related to how older adults and their caregivers viewed sedentary behavior as generally negative but agreed that sitting in a social setting is acceptable. The second theme involved fear of falling and how it is a significant facilitator of sedentary behavior. The final theme revealed discrepant views between residents and staff about how the facility living environment impacts sedentary behavior.

**Theme** **1.** *Older adults and facility staff expressed negative sentiments about sedentary behavior unless it was in the context of social behaviors.*

Participants were asked about how much time they spent sitting each day. Few older adults provided specific estimates of sitting time. Those who did provided estimates of 6 h, 8 h, or 60% of the day. The majority of focus group participants used expressions such as ”too much” or “an awful lot” that provided a level of meaning to how they felt about their daily sedentary time. Drawbacks to sitting included getting stiff, achiness, pain, boredom, and decline in overall health.

Residents discussed sedentary behavior as an inevitable part of aging. As one ILF resident stated:

*I keep moving. But course you get older, and it comes with it. [chuckling] I mean, you know, you don’t want to, but, you know, first chair you find and you take a seat. You know? Like that. It’s old age.*

*(Resident 6 [R6], ILF 1, Group A)*

Discussions of aging were closely integrated with references to physical limitations that many older adults experience, as this exchange between ALF residents illustrates.

*R3: Most of the activities seem to require [us] to sit down, right?*

*R2: Yeah, but we’re all in our 80s and 90s. What else can you do? I mean if you’re here in your 70s or 60s, then you can think about activities that you could do.*

*R1: Well you just have to work at it. That’s what you have to do.*

*(ALF 1, Group A)*

Older adults felt sedentary behavior negatively impacted their mental health. ILF residents in one group discussed how sitting leads to worrying. Residents sought out activities to keep busy and avoid anxious feelings.

*R3: If [I] get a little bored or something, then I walk around or something, because sitting I start to worry too much about things, and I figure the more active you are the less you can worry about things that you can’t do anything about.*

*[others agreeing]*

*R1: It’s true. It’s important to keep busy. If you’re sitting all the time, your mind is wandering where it shouldn’t. You’re thinking about things you shouldn’t be thinking about.*

*R7: You’re thinking about things that you can’t do anything about. You can’t change certain things.*

*R3: Exactly. Absolutely.*

*(ILF 1, Group A)*

Staff members also framed sedentary behavior in the context of aging, referencing physical and mental decline as a consequence of sitting too much.

*But it’s like the, one of the negatives of them sitting down is [residents] kind of just getting older. And that’s mentally draining. Letting your muscles deteriorate is definitely not good.*

*(Staff, ILF 1)*

*And when somebody in their eighties or nineties sits and watches television all day, it’s not good for them.*

*(Staff, ILF 2)*

Older adults and staff both agreed that sitting while either socially or cognitively engaged was distinct from sitting “doing nothing”. Residents discussed seated activities but were clear to emphasize that their minds were also engaged.

*R7: You sit down, you play bingo for an hour or whatever. But you’re doing something when you’re sitting.*

*(ILF 1, Group A)*

*R1: Some activities, you are sitting but you’re not bored…As long as you have something interesting to do, I don’t mind sitting.*

*(ALF 2, Group B)*

When asked directly about the time residents spend sitting, one staff member’s response was framed around the positive effects of social engagement rather than sedentary activities.

*Well, I would rather see them sitting out with a lot of people than sitting by themselves in their room. A lot will come out and, you know, sit in our living room and talk amongst themselves. I like that. We have groups that’ll go out onto the patio and sit on the furniture and talk and just enjoy the weather. Yeah, I like that. You know, I wish everybody would just get out and be more social. But there’s just some people that are just not going to do that.*

*(Staff, ILF 2)*

**Theme** **2.** *Fear of falling is a significant facilitator of sedentary behavior.*

Fear of falling was a prevalent theme discussed throughout the focus groups and interviews. Residents discussed how fear of falling negatively impacts personal motivation to be less sedentary and more active. Many had experienced prior falls that led them to be more sedentary, as described by both ILF and ALF residents.

*R1: Primarily I was much more active when I first came here and then when I had that fall. It’s made me very hesitant.*

*(ILF 2, Group A)*

*R1: When you fall and you’re scared of falling, it limits you. You’re afraid to do things that you probably could do if you weren’t afraid of falling.*

*(ALF 1, Group B)*

One ILF resident described how the motivation to walk and use stairs declined after an injurious fall.

*R2: I know I sit far too much. When we arrived, I walked a great deal. I used the steps, and I walked all three levels and the halls. However, I had a very bad fall off the curb, and hit my face into the street. Now, since then, I haven’t used the steps for fear if I did fall no one would know where I was.*

*(ILF 2, Group B)*

The desire for social support, such as a companion to walk with or a walking group, would help to alleviate some of the fears expressed. The availability of facility staff provided comfort to those concerned about falling alone while walking outdoors.

*R2: I tell the girls at the desk ‘I am going for a walk. If I am not back in the amount of time that usually you think it [takes to go] around—you send somebody out looking for me’ because just—you’re out here and it’s dark and there is no one around. I mean in the front, that’s something else now. I just walk the front of the two buildings. I don’t go around the back any longer.*

*(ALF 2, Group A)*

Fear of falling was also discussed in several interviews with staff. Medications that may cause dizziness and increase the likelihood of falling were mentioned as staff concerns. Several staff described residents with walking difficulties and witnessing prior falls. They felt the fear of falling kept older adults from moving more and participating in activities.

*I know we don’t have much interest in, uh, the exercise class. They like the Tai Chi, but there are only a couple of people who signed up for the exercise class. Like I said, I think mostly it’s because they’re afraid of falling.*

*(Staff, ILF 2)*

ILF and ALF staff felt that one benefit to residents sitting was limiting the risk of falls. Staff in both types of facilities were concerned about resident safety and the risk of injurious falls when residents were standing and walking.

*But the positives of them sitting is them lowering the risks of falling, hurting themselves, straining a muscle, breaking a leg or an arm.*

*(Staff, ILF 1)*

*So as much as I can get them to stand up and do different things, I encourage that. The fear is that you want to be by each one as they do it because, again, just when they stand up, they tend to totter and the chance of, you know, going too far one way and then continuing that way onto the floor outweighs the risk of them just staying in their chair and not doing it.*

*(Staff, ALF 2)*

**Theme** **3.** *While residents of both facility types believed the living environment contributes to their sedentary time, staff felt the facility environment did not promote sitting.*

Residents described their lives as more sedentary after moving into the residential facility.

*R6: Sitting didn’t start ‘til we came here, about three months or something.*

*(ILF 1, Group A)*

Part of the reason sedentary time increased was attributed to the amenities and services provided by facilities, reducing household tasks that offered opportunities for daily light activity.

*R7: When you live in your own home, you have things to take care of. All the time, you have to do this and do that. Here, because we have so much time on our hands, that we don’t have to do cleaning, we don’t have to do dishes, we don’t have to do cooking, our life has changed. And I think everybody would feel the same. So, you know, we were always busy. You know, home to take care of. You had everything to do. But being here is a totally different life.*

*(ILF 1, Group A)*

One facility amenity in particular, the group meal structure, was widely discussed in all focus groups. The influence of mealtimes on sedentary behavior was a topic of debate. In all participating facilities, meals are held in a communal dining room at specific times. Residents are seated and served by staff. Some residents described mealtimes as the longest uninterrupted bouts of sitting they had throughout the day.

*R7: Our meals are an hour and a half, so that’s a long period of time for us to be sitting. You know, the service and everything is great. But it takes long for us to be sit—to be served from the beginning to the end. And so I think that is a long period of time that we all sit, that we maybe normally wouldn’t sit, to eat your meal.*

*(ILF 1, Group A)*

Others viewed meals as an incentive to leave their room and walk to the dining room, as expressed by one ALF resident.

*R2: I think we move around a little bit better with our new social director. She has a lot of activities, but of course when you get there you have to sit too you know again but it’s getting up and down and going on the elevator and walking. So it’s getting you moving a little bit and 3 times a day going to breakfast, lunch, and supper. And I think it’s a little bit better now since we have more activities.*

*(ALF 1, Group A)*

In addition to providing assistance with household chores and meal service, all facilities had an activities director who planned daily events and coordinated outings. Residents discussed several kinds of activities offered by facilities including various forms of exercise (e.g., Tai Chi, Zumba), games, and entertainment. Many of these activities required residents to be seated for an hour or more as the preceding quote illustrates.

Residents also discussed the physical environment and how it influenced their desire to be more active, as this exchange between assisted living facility residents illustrates.

*R2: Well, I’m not too crazy about walking around on the outside here. There is [sic] too many pebbles and there are too many dips [in the sidewalk]. But that’s character of what they need here.*

*R5: Well then do it [walking] on the inside.*

*R2: Oh where?*

*R5: Do it on the floors on the inside, walking around them.*

*R2: That’s true but sometimes you want to get a fresh breath of air.*

*(ALF 2, Group A)*

Another focus group participant suggested the facility post signs indicating the length of hallways as a way for residents to track their progress while walking.

*R2: Could you map out how long from the door down to the end of the hallway, and then back again? You could do your exercise walking if you knew how far you were going. So it would be like ten times down, five times down and back, whatever, you’d be getting some exercise. And I could take my cane. I could walk that hallway. I could come back.*

*R1: Oh, I see what she means.*

*R2: I could go twice, maybe tomorrow I could go three times, the next day four. So if you could—and it’s not going to cost anything to map out the hallway.*

*(ALF 2, Group B)*

While walking was the predominant physical activity referenced across focus groups, residents of one ILF discussed how availability of exercise equipment would provide motivation to be active even for those who have difficulty walking.

*R3: That’s why we’re trying to get a gym here, to motivate the people that think they can’t walk. And no matter how little you can walk—I mean everybody can’t walk with not having a problem—but if we try to motivate these people to do a little bit at a time, and build themselves up… So when we get this gym, for the—our— that should help a lot of people that don’t do anything right now.*

*(others agreeing)*

*R7: It would take away from their sitting time.*

*R3: Right, right.*

*(ILF 1, Group A)*

Staff differed from residents in their view of how the facility living environment influences sedentary behavior and did not endorse the idea that the community living environment promoted more sitting compared to the individual home environment. As one interviewee stated,

*I’m just not sure that if they were living in their own homes that they’d be doing any more walking than they do right now.*

*(Staff, ILF 2)*

Caregivers felt the expectation that older adults can be sitting less is not realistic given the varying levels of physical function among residents. The level of care provided by staff depended on their facility type, which influenced how much they felt they could encourage less sitting among residents. Several staff at ILFs discussed their limited ability to get residents up and moving. As one staff member stated:

*But with us being, you know, independent living, you know, our hands are tied. We can suggest it, but we can’t make anybody do anything.*

*(Staff, ILF 2)*

ALFs by nature provide a higher level of care for residents. Staff are able to provide the physical support necessary for residents with limited physical function to be less sedentary.

*Anytime I can get them to transfer—just that, I mean if they do that—if you figure they go to breakfast, lunch and dinner, that’s, that’s 2, 4, 6 times in and out of the chair there.*

*(Staff, ALF 2)*

When asked about how their facility could encourage residents to sit less, several staff felt there was nothing they or the facility could do to promote less prolonged sedentary periods. Rather, they consistently described the variety of physical and social activities offered to residents by the facility—even though most involved long bouts of sitting—asserting it was a matter of autonomy and motivation on behalf of the residents to sit less.

*Firstly, I don’t know any place that presents more things to do that are not unreasonable, you know, things that they are not capable of doing, than where I work. … You can’t make people do what they don’t want to do. But you can certainly encourage and have enough things out there, and try to, you know, motivate. And I believe that this takes place all the time.*

*(Staff, ILF 2)*

*Well, from what I’ve seen, [residents] lack the initiative to get involved. … I mean, we try to encourage them. But, you know, it’s entirely up to them. And some people, you know, are motivated, and other ones just - they’re happy and contented just sitting, you know, and chatting, you know, with their friends.*

*(Staff, ILF 1)*

For residents with limited physical function, availability of staff at ALFs to provide physical aid was a barrier to improving resident sedentary behavior. As one caregiver stated:

*We could probably use more people in Activities. I am the only person here. That with our—the mobility that we have with our residents, you need one on one.*

*(Staff, ALF 2)*

## 4. Discussion

Our findings provide new insights into how older adults in residential communities view sedentary time and the influence of the living environment on their sedentary behavior. We identified three key themes describing factors at several levels of the Social Ecological Model that shape sedentary behavior in this older adult population. We found similar themes emerge from resident focus groups across independent and assisted living facilities, reflecting the relative similarities of the participants (Table 1). Residents and staff recognized the negative impact of sitting on physical and mental well-being, while acknowledging the social and cognitive benefits of seated activities and the physical limitations to sitting less. Fear of falling was widely discussed as a significant motivator for older adults to remain seated. This fear was influenced by the availability of social support and the physical environment of the living community. Older adults felt they had become more sedentary after moving into the residential community, although staff felt the facility living environment did not encourage more sedentary behavior. Our results provide new information necessary for intervention development specifically for this highly sedentary population of older adults in residential care communities.

Our first theme captured perceptions of sedentary behavior held by ILF and ALF residents and staff. Older adults felt that increased sitting was a natural consequence of aging, yet they placed judgment on the amount of time they spent sitting, illustrating an understanding that prolonged periods of sitting are not good for their health. Residents and staff both mentioned physical health, motivation, and safety concerns as significant barriers to sitting less. Our findings are similar to those reported in community-dwelling older adults. In their thematic analysis, Compernolle et al. found that physical limitations impact motivation to participate in non-sedentary activities [12]. Similarly, health status was found to be a barrier to physical activity in long-term care [26]. We found that declining physical function was closely integrated with mentions of age and aging and was used by both residents and staff to justify sitting for long periods. Interventions to change sedentary behavior in these settings would need to address varying physical abilities as many participants simply stated they were unable to exercise or walk due to limited physical ability. Even in the context of independent living communities, residents differed in their level of function with some able to walk independently and others who relied on an assistive device for mobility. The distinction between independent and assisted living depends on the level of care offered by the facility staff and is not necessarily indicative of the functionality of the residents. For example, those in ILF needing a higher level of assistance may choose to stay in the ILF setting and receive help from family or friends rather than move to an ALF. Older adults with walking difficulties may view an intervention targeting breaks in sedentary time as more acceptable than one encouraging walking. Increasing the number of daily sit-to-stand transitions can be an intervention goal distinct from increasing physical activity [27]. Standing breaks, short walks, or movement to disrupt long bouts of sedentary behavior may be viewed as more attainable goals for this population compared to an intervention to promote more walking.

Older adults and staff agreed that sitting and being socially and/or cognitively engaged was more acceptable than sitting alone. Our findings echo those of others who have found community-dwelling older adults describe engaging in sedentary activities as positive due to the social and cognitive nature of the activities [28,29]. Interventions to change sedentary behavior in the ILF or ALF setting will need to include education for both residents and staff about the negative physiologic effects related to extended sitting, even if socially engaged. Seated activities such as talking with friends and reading are important for psychosocial health and should not be discouraged outright [30]. Rather, prolonged bouts of sitting during these beneficial activities could be interrupted to combat the negative physiologic impact of extended sitting. Given the existing facility infrastructure that supports social amenities, social activities are an ideal target for reducing sedentary behavior at the facility level as they also provide a social support network for behavior change [17,31]. This particular strategy may prove effective given that participants in our study mentioned accumulating long bouts of sedentary time during facility-sponsored activities. However, a recent pilot study incorporating a similar approach as part of a multicomponent intervention reported lack of follow through by facility staff to prompt breaks during activities and stressed the importance of staff engagement for successful implementation of this approach [32].

Fear of falling was a significant facilitator of sedentary behavior that was discussed in nearly all focus groups. This concept traversed multiple levels of the Social Ecological Model, impacting personal motivation, underlying the desire for social support, and prompting suggestions for changing the physical environment. Some older adults did not feel safe walking outside of the facility due to environmental hazards. Residents were comforted by having a walking partner or a system in place where their whereabouts were known during walking in case of an adverse event. Implementing a system at the facility level, particularly in ILFs, for residents to notify staff when they are walking outside may help to alleviate resident fears and provide more opportunity for activity. Staff agreed that too much sedentary behavior is not good for residents; however, they also viewed sitting as beneficial in that it enhances resident safety. These findings are similar to a study of sedentary behavior among Scottish older adults that found family members often encourage older adults to sit to prevent injury [19]. The belief that sitting is safer than standing or walking may lead staff to indirectly facilitate more resident sitting [26]. Providing residents and staff instruction on safe ways to interrupt sedentary behavior, such as standing with arm support, may help reduce fear of falling and lessen prolonged bouts of sedentary behavior.

Our most novel finding was how the facility living environment influences sedentary behavior of older adult residents. Several focus group members discussed being more sedentary after transitioning from community living to a planned residential facility. With daily household tasks such as cooking and light cleaning provided by the living facility, older adults reported that they became much more sedentary. Previous research identified household activities as a key domain of activity in daily life [11,29,33]. The household domain has been suggested as a primary target for changing sedentary behavior, increasing daily light household activities and breaking up extended periods of sitting with chores [29]. While this is a reasonable target for older adults living in the community, our research suggests these types of interventions are likely to be less effective among those in residential care facilities who have lessened household responsibilities and limited opportunities to perform routine household chores. Instead, our findings suggest social activities may be a primary target for change. Residents often sat for extended periods during facility-sponsored activities like games and exercise as well as during mealtimes. Incorporating standing breaks as part of the group activities could be one strategy implemented at the facility level to intervene on prolonged sedentary behavior of residents. Interventions such as this would have to incorporate facility-level changes, requiring institutional support from administration and staff. Importantly, we also discovered that staff may not realize how the living environment influences resident behavior, particularly in ILFs. Organizational readiness for change [34] would need to be addressed to enhance success of a systems-level sedentary behavior intervention in the living facility setting. Involving residents and staff in the creation of the intervention may improve acceptability and feasibility of implementation as has been recently demonstrated by the GET READY Study [35].

Our work corroborates and expands on previous studies that have used the Social Ecological Model to frame determinants of sedentary behavior in older adults. Individual level factors such as health status, pain, fatigue, and motivation, as well as social level factors including the need or desire for support in reducing sedentary behavior have been expressed by both community-dwelling older adults [20] and assisted living residents [36]. While individual and social level factors are common between these populations, the residential living environment presents new influences on sedentary behavior that have not been fully explored. Recently, Voss et al. published findings from focus groups with assisted living facility residents across six care homes in Canada [36]. They identified additional factors unique to the residential care environment that facilitate sedentary behavior in this population. At the organizational level, residents described how the lack of sponsored activities outside of business hours leads to more sedentary behavior during evenings and weekends. Similar to our findings, participants also referenced how the living environment led to a decrease in daily household activities and engagement in more sedentary activities compared to living in their own home [36]. We provide additional context surrounding the interpersonal relationships between staff and residents and how staff attitudes and behaviors shape the facility culture and contribute to resident sedentary behavior. While residents acknowledged both personal factors and factors of the living environment that influence their sedentary behavior, facility staff put the onus to be less sedentary on the individual resident. Several staff referenced organizational programming offered for residents and did not recognize that these activities were primarily sedentary, even those involving exercise such as chair-based workouts and seated yoga. It is important for intervention design to consider the specific context and setting in which sedentary behavior occurs [11] and target behavior change at multiple levels of the Social Ecological Model. Additional exploration of the extent to which staff attitudes and organizational policies influence resident sedentary behavior is warranted.

Our study does have limitations. The views expressed by focus group and interview participants may have limited generalizability to populations different from those included in the study. We stratified focus groups by living facility type and were not able to make comparisons among older adults based on gender or age groups. In their examination of older adults in retirement communities, Belletierre et al. demonstrated gender and age differences in sedentary behavior patterns [37]. Future studies may wish to explore how older adults’ perspectives on sedentary behavior differ based on these characteristics. We reached thematic saturation among focus group participants but not with interviewed staff. Staff recruitment did not yield the intended target despite extensive recruitment efforts, potentially due to discomfort discussing topics related to their workplace and the facility residents. Although telephone interviews offer several advantages (e.g., anonymity, privacy) [38,39,40], we were unable to observe body language that may have conveyed discomfort on the part of staff. We were unable to make comparisons between staff at the different facility types due to the small number of staff interviewed. Additionally, there may be more topics brought forth by staff working in these or other types of residential facilities regarding resident sedentary behavior that were not discussed in the interviews conducted.

Our study also had several strengths. We sought the perspectives of both older adults and caregivers, allowing us to understand when viewpoints about sedentary behavior converged (e.g., fear of falling) and when they differed (e.g., the influence of the living environment). We revealed environmental influences that may not impact those living independently in their own home and identified opportunities for intervention unique to the living community setting such as facility-sponsored social activities. Our sample included participants from ILF and ALF settings, providing much needed information for development of systems-based interventions to reduce sedentary behavior among older adults across different levels of care.

## 5. Conclusions

Our results reveal how the social and physical living environments of ILF and ALF communities affect sedentary behavior in older adults. We provide valuable information about resident and staff perspectives on sedentary behavior needed for designing interventions tailored to older adults in residential living facilities. Most prior studies have focused on community-dwelling older adults and individual-level factors influencing sedentary behavior [10]. The ILF and ALF living environments represent close communities of older adults at high risk for negative health outcomes related to excessive sedentary behavior. Changing structural factors that either directly or indirectly impact individual behavior may be a more fruitful approach in this population of older adult at high risk for sedentary behavior [14]. Our findings suggest thinking beyond the individual when seeking to intervene on sedentary behavior and considering the unique influences of the residential living environment. We found that staff emphasize individual abilities and attitudes and failed to recognize how the facility culture also impacts resident sedentary behavior. Further research into the perspectives of staff and facility leaders is needed to identify and address organizational barriers to implementing facility-wide interventions.

## Figures and Tables

**Table 1:** Focus Group Participant Characteristics.

	ILFs (*n* = 22)	ALFs (*n* = 22)	Total (*n* = 44)
Age, mean years (range)	86 (65–93)	86 (65–97)	86 (65–97)
Female, *n* (%)	14 (63.6)	17 (77.3)	31 (70.5)
Race, *n* (%)			
White	22 (100)	20 (90.9)	42 (95.4)
Not provided	0 (0.0)	2 (9.1)	2 (4.6)
Marital status, *n* (%)			
Never married	1 (4.5)	2 (9.1)	3 (6.8)
Married	7 (31.8)	2 (9.1)	9 (20.5)
Divorced	1 (4.5)	2 (9.1)	3 (6.8)
Widowed	13 (59.1)	16 (72.7)	29 (65.9)
Assistive device use ^1^, *n* (%)	13 (59.1)	19 (86.4)	32 (72.7)
Fall in past year ^2^, *n* (%)	8 (36.4)	10 (45.5)	18 (40.9)
Physical activity ^2^, *n* (%)			
Daily	7 (31.8)	9 (40.9)	16 (36.4)
Few days per week	5 (22.7)	7 (31.8)	12 (27.3)
Less than once per week	4 (18.2)	2 (9.1)	6 (13.6)
Never	3 (13.6)	2 (9.1)	5 (11.4)
Not provided	3 (13.6)	2 (9.1)	5 (11.4)

^1^ Assistive devices included canes, walkers, rollators, wheelchairs, and motorized scooters. ^2^ Based on participant self-report. ILF = Independent living facility; ALF = Assisted living facility.

## Supplementary Materials

The following are available online at https://www.mdpi.com/1660-4601/17/17/6415/s1, S1: Resident Focus Group Guide, S2: Staff Interview Guide.
