# Supplementary material for: Personal and Environmental Contributors to Sedentary Behavior of Older Adults in Independent and Assisted Living Facilities
Source: Int J Environ Res Public Health. 2020 Sep 3;17(17):6415. doi: 10.3390/ijerph17176415 (PMC7504320; doi:10.3390/ijerph17176415)
Supplement: Supplementary file 1 [file ijerph-17-06415-s001.zip › Supplemental File 2.pdf]

Supplemental File 2: Staff Interview Question Guide

What is your role at your facility, and how long have you been working in it? Have you worked with older adults in long-term care before? If so, how long have you cared for older adults? What types of settings have you worked in previously?

How much time do you think the residents at your facility spend sitting on a typical day? (If it varies, what is the range?)

Why do you think some residents might sit for longer periods of time compared to others?

What activities do residents at your facility do that require sitting? What about activities that require standing or walking?

How do you feel about the amount of time that residents spend sitting? How would you feel about encouraging them to sit less?

Does your facility have any formal policy or preferences on resident sitting, or on encouraging residents to stand/walk?

Do your residents or their families have any preferences regarding how long they sit?

What are the benefits to having residents sit? What about drawbacks to having them sit?

If you wanted residents to sit less, what problems might you face? Are there barriers to their standing/walking more?

What could your facility do to promote less sitting for extended periods among residents?

Probe if necessary: changes in physical environment, activities/programming, policies?
