# Supplementary material for: Personal and Environmental Contributors to Sedentary Behavior of Older Adults in Independent and Assisted Living Facilities
Source: Int J Environ Res Public Health. 2020 Sep 3;17(17):6415. doi: 10.3390/ijerph17176415 (PMC7504320; doi:10.3390/ijerph17176415)
Supplement: Supplementary file 1 [file ijerph-17-06415-s001.zip › Supplemental File 1.pdf]

Supplemental File 1: Focus Group Question Guide

First, I'd like us all to introduce ourselves. Let's go around the room, and share your first name only, how long you've lived here, and your best guess about how much time you spend sitting on a regular day.

How do you feel about how much time you spend sitting? Is it too much, not enough? How much is too much?

When do you feel you are sitting for the longest stretches of time? What activities do you do that require sitting?

When do you feel that you are standing or walking for the longest stretches of time? What activities do you do that require standing or walking?

Which type of activities do you prefer – those that require you to stand or walk, or those that require you to sit – and why?

Have your preferences for these activities, or for sitting or standing, changed at all as you've aged? How so?

What are some of the benefits that you see to sitting?

What about drawbacks? What are the drawbacks to sitting?

What prevents you from getting up and sitting less?

What do you think that [name of facility] thinks about your sitting? Do they encourage you to sit? Do they encourage you to get up? What do they do that encourages you to sit or get up?

How does the living environment here influence the amount of time you spend sitting? Do you feel it encourages you to sit more or less?

What changes to the living space might help you to sit less?

Do your friends or family have anything to say about your sitting? What have they told your or said?

If you wanted to set less during the day, what problems might you face?

Lastly, what, if anything, might encourage you to get up more often?

> Probe if necessary: More information on the impact of sitting? Encouragement from others? Different activities?
